# Catalytic Hydrogenation of CO_2_ to Alternative Fuels: A Review of Methanation and Related Pathways

**DOI:** 10.3390/ma19163541

**Published:** 2026-08-21

**Authors:** Kornelia Nejranowska, Agnieszka Szymaszek-Wawryca, Monika Motak

**Affiliations:** Faculty of Energy and Fuels, AGH University of Krakow, 30-059 Krakow, Poland; agnszym@agh.edu.pl (A.S.-W.); motakm@agh.edu.pl (M.M.)

**Keywords:** CO_2_ hydrogenation, Carbon Capture, Utilization, and Storage (CCUS), methanation, RWGS, heterogeneous catalysis, sustainable aviation fuels (SAF)

## Abstract

The imperative to mitigate climate change has accelerated the development of Carbon Capture, Utilization, and Storage (CCUS) technologies, particularly CO_2_ hydrogenation into high-value chemicals and alternative fuels. This work evaluates the fundamental thermodynamic limitations and the primary directions of CO_2_ conversion, with a primary focus on methanation, alongside related pathways such as methanol synthesis and the reverse water-gas shift (RWGS) reaction. To overcome the high kinetic barriers of CO_2_ activation, various catalytic systems are analyzed. While noble metal catalysts exhibit high catalytic performance, nickel-based catalysts serve as a viable and cost-effective alternative. To overcome nickel’s susceptibility to thermal sintering and coking, advanced bimetallic and multimetallic formulations are being developed to enhance structural stability and selectivity. These advancements are crucial for producing Synthetic Natural Gas (SNG) and sustainable aviation fuels (SAF). Ultimately, the objective of this comprehensive review is to systematically summarize recent advancements in catalyst design, critically analyze the advantages and fundamental bottlenecks of distinct catalytic systems, and outline prospective paths for the efficient industrial-scale production of sustainable alternative fuels.

## 1. Introduction

### 1.1. The Importance of Carbon, Capture, Utilization and Storage (CCUS)

Carbon dioxide (CO_2_) emissions have been a key factor driving climate change over the past few decades. The increase in the concentration of this gas in the atmosphere intensifies the greenhouse effect, which results from its ability to absorb and re-emit infrared radiation from the Earth’s surface. This phenomenon directly contributes to an increase in the frequency of weather anomalies, droughts, and floods, and also has a significant impact on the stability of environmental and hydrological systems [1]. In the face of challenges posed by the increasing carbon dioxide emissions, the role of CO_2_ as a raw material in industrial processes is becoming increasingly important. The concept of Carbon Capture, Utilization, and Storage (CCUS) facilitates a shift away from treating CO_2_ solely as waste, focusing efforts on converting it into high-value-added products. A key focus of CCUS is the permanent sequestration of carbon dioxide in the form of stable chemical compounds. Consequently, emissions are effectively reduced, while simultaneously managing industrial waste. However, the overall efficiency of the CCUS value chain relies heavily on the initial carbon capture step. While conventional amine scrubbing (e.g., monoethanolamine, MEA) remains the industrial standard, it suffers from high toxicity, volatility, and massive regeneration energy penalties. To address these limitations, highly promising “green solvents” are being developed, such as alkaline potassium carbonate solutions promoted with amino acid salts (e.g., sarcosine, glycine, or alanine). These chemical promoters significantly accelerate CO_2_ absorption, achieving loading capacities comparable to conventional amines, but with high biodegradability and drastically reduced energy requirements for solvent regeneration [2]. Developing advanced catalytic systems with tailored supports opens new pathways for efficiently converting CO_2_ into valuable chemicals and fuels [3].

### 1.2. Thermodynamic and Kinetic Aspects of CO_2_ Hydrogenation

The activation of CO_2_ is highly challenging because the molecule is both thermodynamically stable and kinetically inert. From a thermodynamic perspective, CO_2_ represents a stable, low-energy state, reflected by its highly negative standard enthalpy of formation (−393.5 kJ mol^−1^) and Gibbs free energy (−394.4 kJ mol^−1^). Furthermore, the carbon atom in CO_2_ is in its highest oxidation state (+4), which significantly limits its reducibility [4]. Conversely, from a kinetic perspective, the CO_2_ molecule is highly inert due to its linear geometry and the presence of strong C=O double bonds. Therefore, overcoming this high kinetic activation energy barrier necessitates the use of highly efficient catalytic systems [5].

To overcome the aforementioned thermodynamic limitations, CO_2_ must interact with compounds characterized by high Gibbs free energy, such as H_2_ (0 kJ mol^−1^). This interaction is illustrated by the direct hydrogenation of CO_2_ with H_2_ to form alkanes, as shown in Equation (1) [6].

n CO_2_ + (3n + 1) H_2_ ⇔ C_n_H_2n+2_ + 2n H_2_O (1)

Because multiple pathways occur simultaneously during CO_2_ hydrogenation, thermodynamic calculations are critical for predicting compound stability, reactant conversion, and product selectivity. These factors may be influenced by changes in reaction parameters, such as temperature, pressure, and reagent ratios. To predict the thermodynamic behavior, expected reactant conversion, and product selectivity, the standard enthalpies and Gibbs free energies for key CO_2_ hydrogenation pathways are summarized in Table 1 [6].

As demonstrated by the data in Table 1, the thermodynamic profiles of these specific pathways differ significantly. For instance, CO_2_ methanation is highly exothermic and thermodynamically favorable (ΔG° < 0), even at standard conditions. In contrast, the reverse water-gas shift reaction leading to carbon monoxide is endothermic (ΔH° > 0) and requires significantly elevated temperatures to shift the equilibrium toward the products. Conversely, methanol synthesis is moderately exothermic but faces equilibrium limitations at higher temperatures, necessitating carefully optimized conditions, typically lower temperatures and higher pressures, to maximize the yield [6].

### 1.3. Literature Selection Methodology

To ensure a structured and comprehensive evaluation of recent advancements in CO_2_ hydrogenation, a systematic literature search was conducted using the ScienceDirect and Google Scholar databases. The primary search covered articles published between 2021 and 2026 to capture the most recent technological breakthroughs. The search strategy utilized combinations of the following keywords: “CO_2_ methanation,” “reverse water-gas shift,” “CO_2_ to methanol,” “sustainable aviation fuels,” and “catalyst deactivation.” The retrieved publications were initialy screened based on their direct relevance to heterogeneous catalytic CO_2_ hydrogenation. Emphasis was placed on original research articles and recent authoritative reviews reporting catalyst composition, preparation methods, catalytic performance, reaction mechanisms, and deactivation phenomena. Studies lacking detailed operating conditions or focusing exclusively on homogeneous catalysis were excluded. Ultimately, representative studies were selected according to their scientific rigor, citation impact, novelty, and overall contribution to understanding the current state of the art in CCUS technologies. Following the initial screening of over 150 publications, a final set of 57 core studies was selected for detailed analysis based on their direct relevance to heterogeneous CO_2_ methanation, related hydrogenation pathways, and catalyst deactivation mechanisms.

## 2. Conversion Directions and Competing Reactions

Hydrogen plays a key role in CO_2_ conversion processes, serving as a reducing agent and acting as the primary reagent that enables the conversion of carbon dioxide into valuable chemical compounds [7]. In many technological concepts, the hydrogen used in these processes can be derived from water electrolysis, powered by renewable energy sources, which aligns with the concept of a low-carbon economy and the reduction in greenhouse gas emissions. This concept is known as power-to-gas (PtG) and involves converting electricity into gaseous fuels, such as hydrogen or methane, while using CO_2_ as a feedstock [8].

### 2.1. CO_2_ Methanation

An important example of CO_2_ conversion is the reversible, exothermic Sabatier reaction (Equation (2)), in which carbon dioxide is hydrogenated to methane. This process forms the basis of so-called CO_2_ methanation and takes place on metallic catalysts, most commonly nickel-based ones [9]. Despite its apparent simplicity, this reaction requires precisely optimized thermodynamic and catalytic conditions, often involving elevated temperatures and the presence of an efficient catalytic system [3].

CO_2_ + 4H_2_ ⇔ CH_4_ + 2H_2_O Δ*H* _298 K_ = −165 kJ/mol (2)

Thermodynamically, CO_2_ methanation is most favorable at low temperatures due to its exothermic nature [10]. Practical low-temperature applications face kinetic limitations, whereas temperatures above 450 °C promote the endothermic reverse water-gas shift (RWGS) reaction, reducing CO_2_ conversion and methane selectivity [10,11]. When integrated into industrial Power-to-Gas (PtG) frameworks, methanation offers a promising pathway for carbon recycling by combining captured CO_2_ with green hydrogen generated via renewable-powered water electrolysis [12]. This carbon capture and utilization (CCU) strategy mitigates global warming and promotes circularity [11,13]. The resulting methane can serve as a green feedstock for producing CO_2_-free turquoise hydrogen and solid carbon via catalytic pyrolysis [11,12]. Recently, overcoming the requirement for elevated operating temperatures has become a primary focus of process optimization [10]. Modern optimized catalytic processes have demonstrated the feasibility of achieving CO_2_ conversion rates exceeding 90% with near-complete methane selectivity (up to 99%) at temperatures as low as 250–260 °C. To meet the rigorous productivity demands of industrial scaling, recent advancements have also shown that robust conversion rates can be sustained under considerable high gas hourly space velocities (GHSV) exceeding 50,000 mL g^−1^ h^−1^ [11]. Managing such high flow rates while maintaining rapid reaction kinetics is a critical design variable for scaling up the technology. Despite these process-level achievements, several bottlenecks hinder the transition to full-scale industrial operations. Lowering the reaction temperature exacerbates kinetic limitations, necessitating highly loaded or complex catalyst architectures, which significantly increases overall synthesis and materials costs [10,11]. Furthermore, continuous industrial operations are highly susceptible to catalyst deactivation through carbon deposition (coking) and thermal sintering [10]. Finally, raw CO_2_ feeds often contain trace impurities, such as hydrogen sulfide (H_2_S), which strongly chemisorb onto active metallic surfaces, blocking active sites, promoting metal oxidation, and accelerating irreversible catalyst deactivation and metal sintering [10]. A detailed analysis of the specific catalytic materials and advanced supports engineered to overcome these process limitations is presented in Section 3.

### 2.2. Methanol Synthesis

Another possible product of CO_2_ hydrogenation is methanol (Equation (3)) [14,15]. It can be used both as an alternative fuel and as a feedstock for many chemical syntheses, making it of particular importance to the chemical industry.

CO_2_ + 3H_2_ ⇔ CH_3_OH + H_2_O Δ*H*° _298 K_ = −49.6 kJ/mol (3)

The efficiency of this intensively studied process depends heavily on supported heterogeneous catalysts, which must possess several key physicochemical properties to guarantee optimal activity, selectivity, and long-term stability. First, the catalyst must provide active sites capable of efficiently adsorbing and activating the highly stable CO_2_ molecule, while simultaneously facilitating hydrogen dissociation [16]. The catalyst surface must promote the formation and transformation of reaction intermediates, such as formate and methoxy species, which are widely recognized as crucial intermediates in the methanol synthesis pathway [16,17]. Furthermore, an appropriate balance between acidic and basic surface sites is required to enhance CO_2_ adsorption and intermediate stabilization without promoting undesirable side reactions [18]. High metal dispersion and a large specific surface area are also desirable, as they increase the number of accessible active sites. Equally important is the presence of strong metal-support interactions, which can modify the electronic properties of the active phase, improve catalyst stability, and suppress sintering under reaction conditions [19]. Finally, the catalyst should exhibit high selectivity toward methanol formation while minimizing competing reactions, particularly the reverse water-gas shift reaction, which leads to CO production and decreases methanol yield [20]. Recently, breaking the thermodynamic and kinetic barriers of low-pressure CO_2_ hydrogenation to methanol has yielded significant process-level results [10]. Modern catalytic systems operate efficiently at low temperatures (175 °C) and atmospheric pressure, achieving methanol yields up to 86.31 mmol g^−1^ h^−1^ with 40% CO_2_ conversion and 88% selectivity. Concurrently, other ambient-pressure configurations have demonstrated methanol space-time yields (STY) of approximately 0.67 mmol g^−1^ h^−1^ at 240 °C [21,22]. Optimized dual-site configurations achieve 100% methanol selectivity at 3.0 MPa and 270 °C. This maximizes yield (up to 2500 mg CH_3_OH gCu^−1^ h^−1^) by completely suppressing the RWGS pathway [10]. To enhance process economics and reduce energy penalties from separate CO_2_ capture and transport, recent studies explore integrated CO_2_ capture and utilization (ICCU) using carbon-supported dual-functional materials [13]. The economic viability of this synthesis remains closely linked to the availability of low-cost green hydrogen, since water electrolysis remains highly energy-intensive (~285 kJ/mol-H_2_) and costly [12]. It is highlighting the need for comprehensive carbon capture and utilization (CCU) strategies to mitigate global warming [11,13].

Despite these process optimizations, scaling up direct CO_2_-to-methanol technology remains limited by fundamental bottlenecks. From a thermodynamic perspective, operating at atmospheric or low pressures, while drastically reducing industrial compression costs, severely limits the overall CO_2_ equilibrium conversion. Moreover, the substantial amount of water (H_2_O) generated as a major byproduct (Equation (3)) can competitively adsorb on active sites, potentially decreasing catalytic efficiency depending on the hydrophobicity of the support during continuous operations, particularly under high-pressure conditions and at high CO_2_ conversion levels [10]. The severity of this effect strongly depends on the specific catalyst composition, operating conditions, and the hydrophobicity of the support material. Water vapor competes for active adsorption sites, accelerates thermal sintering of mobile metal phases like copper [10,11] and induces structural and oxidative deactivation of catalyst supports. Carbon-based matrices are particularly susceptible to such oxidative degradation in water-rich environments [11]. Finding an optimal balance between process conditions, reactant utilization, and acceptable catalyst lifetime remains a critical challenge for industrial CCUS scalability [10,11].

### 2.3. Sustainable Aviation Fuels

The culmination of catalytic CO_2_ conversion is the production of strategic energy carriers, including synthetic natural gas (SNG) and sustainable aviation fuels (SAF) [23]. This approach addresses the problem of greenhouse gas emissions while simultaneously satisfying the global fuel demand [24]. Aviation represents a particular challenge for decarbonization. Due to the requirement for high energy density in long-haul flights, the full electrification of aircraft is unfeasible in the near future [25]. This sector requires liquid “drop-in” fuels, which are synthetic alternatives that can be introduced into the tanks of existing aircraft and pumped through current infrastructure without any modifications [26]. Currently, the catalytic synthesis of SAF from captured CO_2_ is pursued through several distinct technological pathways:

- RWGS–Fischer-Tropsch (FT) route: The most technologically mature two-step process. It involves the initial conversion of CO_2_ to carbon monoxide via the endothermic RWGS reaction, followed by hydrocarbon chain growth through FT synthesis. The FT step typically utilizes established cobalt- or iron-based catalysts operating at high pressures, favoring the production of linear alkanes [24].
- Methanol-to-Jet (MtJ) route: An emerging alternative where CO_2_ is first hydrogenated to methanol (e.g., over Cu/ZnO/Al_2_O_3_), which is subsequently oligomerized into jet-range olefins and aromatics. This conversion is driven by acidic solid catalysts, such as modified ZSM-5 zeolites. Importantly, this route inherently yields higher selectivities toward branched iso-paraffins and aromatics compared to the standard FT process [27,28].
- Tandem hydrogenation: A developing approach focusing on direct, single-step CO_2_ hydrogenation to jet-fuel range hydrocarbons. This relies on multifunctional tandem catalysts [25,29]. This approach aims to bypass intermediate isolation steps, overcoming the thermodynamic limitations of individual reactions and reducing overall capital costs [30,31].

Regardless of the primary synthesis pathway, the raw synthetic crude typically requires specific upgrading routes. For instance, the raw fuel from the FT reactor is almost exclusively paraffinic and virtually aromatic-free. In aviation, aromatics are generally required because they induce the swelling of elastomeric seals, thereby preventing fuel leaks. However, rather than being strictly mandatory for combustion, their presence is dictated by international blending requirements for drop-in synthetic e-fuels, where aromatic content is typically capped between 8% and 25% [24,26]. Therefore, the raw e-fuel must undergo hydrocracking, isomerization, and aromatic alkylation to lower its freeze point and comply with aviation safety specifications [26,27]. Despite the complexity of the process, integrated synthetic refineries offer substantial environmental benefits. When powered by green hydrogen and renewable electricity, they can be thermally self-sufficient, with pure water as their only by-product. An optimally designed process can even ensure a net-negative carbon balance, capable of reducing atmospheric emissions by up to −2 kg of CO_2_ equivalent for every kilogram of fuel produced [28,29]. However, an objective evaluation of this metric requires clearly defined system boundaries for the life-cycle assessment (LCA). This reported net-negative value strictly applies to a ‘cradle-to-gate’ boundary, which accounts for atmospheric or biogenic CO_2_ capture, green hydrogen generation, and fuel processing. It explicitly excludes downstream logistics and final fuel combustion in aircraft engines, which would transition the overall balance toward carbon neutrality [28]. Recent techno-economic analyses indicate that maximizing profitability primarily requires minimizing the consumption of cost-intensive “green” hydrogen, rather than solely reducing electricity demand [32].

### 2.4. Side Reactions and Catalyst Deactivation Pathways

While CO_2_ methanation is a valuable standalone process for producing synthetic natural gas (SNG), in the context of SAF synthesis, it acts as a highly undesirable competing side reaction. Because the RWGS reaction (Equation (4)) is endothermic, industrial processes traditionally operate at elevated temperatures to achieve high equilibrium conversions. However, the exact temperature requirement (often ranging from 400 to over 600 °C) is highly dependent on the specific catalyst composition and operating conditions.

CO_2_ + H_2_ ⇔ CO + H_2_O Δ*H*° _298 K_ = 41 kJ/mol (4)

At lower temperatures (<600 °C), CO_2_ dissociation preferentially leads to methanation. This competing methanation reaction poses a major challenge, as it consumes valuable hydrogen, decreases CO selectivity, and limits the overall efficiency of the downstream FT synthesis. Modern RWGS catalyst engineering focuses on highly active formulations that operate at lower temperatures, saving energy while suppressing CH_4_ selectivity and maximizing CO formation. Mechanistically, this CO_2_-to-CO conversion over advanced catalytic systems (e.g., Cu, Pt, or Ni supported on reducible oxides) is achieved through two primary pathways. The first is the redox mechanism, where the catalyst surface is directly oxidized by CO_2_ to form CO, and subsequently reduced back by H_2_ [33]. The second is the associative pathway (based on the Langmuir-Hinshelwood model), which involves the co-adsorption of CO_2_ and H_2_ on the catalyst surface to form intermediates such as formates, carboxyl groups, and carbonates, which eventually decompose into CO and water [34].

Under specific unoptimized conditions, long-term operation frequently leads to deactivation primarily through coking. This process is typically favored at 300–450 °C, but its extent is heavily dictated by the specific active metal composition and the prevailing H_2_/CO_x_ ratio. Particularly under hydrogen-deficient conditions, carbon predominantly deposits as a result of carbon monoxide disproportionation through the highly exothermic Boudouard reaction (Equation (5)) [35].

2 CO → CO_2_ + C (5)

The deactivation mechanism here is extremely aggressive: the forming carbon diffuses through the crystal lattice of the active metal nanoparticles and grows away from the catalytic sites in the form of carbon nanotubes or filamentous coke. Because filamentous coke forms away from the active sites, it does not immediately block access to them. However, these structures grow with such substantial mechanical force that they physically push and detach the metal crystallite from the support (causing metal-support separation), ultimately destroying the catalyst’s architecture [36]. In contrast, the direct cause of complete and irreversible activity loss is encapsulating coke. It directly covers the metal center, creating a tight shell that completely isolates the active particle from the reaction environment [37].

## 3. Catalytic Reactions in CO_2_ Hydrogenation Processes

As discussed in previous sections, increasing CO_2_ emissions represent one of the greatest challenges of the modern world. Utilizing CO_2_ as a feedstock for alternative fuel production perfectly aligns with decarbonization strategies. However, because the CO_2_ molecule is notable stable both thermodynamically and kinetically, driving these conversion reactions at acceptable rates requires highly specialized materials. The following subsections systematically review advancements in heterogeneous catalyst design, encompassing conventional nickel, noble metals, and advanced bimetallic and multimetallic configurations. Table 2 summarizes the main advantages, limitations, and fundamental bottlenecks of the representative catalyst categories discussed in this section.

### 3.1. Noble Metal Catalysts

Catalysts based on noble metals (e.g., Ru, Rh, Pt, Pd) are widely recognized for their substantial catalytic activity and high hydrogen dissociation capability [40]. However, despite these advantages, their high cost remains a significant barrier to large-scale industrialization. Furthermore, noble metals are often susceptible to thermal sintering under operating conditions and typically require the addition of specific promoters or advanced support architectures to prevent the agglomeration of the active phase [38]. The reaction pathway and selectivity depend critically on the specific noble metal used. The turnover frequency (TOF) for CO_2_ conversion on titanium dioxide (TiO_2_) increases in the order Pd < Pt < Ru < Rh [40]. Importantly, ruthenium and rhodium show very high selectivity toward methane, while platinum and palladium preferentially promote the formation of carbon monoxide via the RWGS reaction [38]. A highly significant mechanistic discovery is the strong structure sensitivity of the methanation reaction on ruthenium catalysts [39]. For instance, studies on Ru/TiO_2_ catalysts under continuous methanation show that catalytic activity significantly increases with Ru crystallite size. Larger Ru particles provide specific multi-atom surface arrangements. These arrangements facilitate the formation of key reactive surface intermediates, which are specifically referred to as Ru_x_-CO structures. The presence of these multi-atom active sites is crucial because they effectively bind and weaken the strong C-O bonds, ultimately driving the high methane yield [40]. Because of these economic and operational limitations of noble metals, non-precious metals (such as nickel) are currently utilized as the primary industrial alternative [38,49].

### 3.2. Nickel Catalysts

In industrial practice, nickel is the most frequently used active metal for CO_2_ methanation because it combines relatively high activity with low cost. However, the design of pure nickel-based systems must account for inherent challenges, primarily their high susceptibility to deactivation due to the thermal sintering of nanoparticles and the accumulation of carbon deposits [37]. Furthermore, while uniform dispersion and low-temperature reducibility can be readily achieved with nickel, it requires careful optimization of the support and synthesis method to prevent active phase agglomeration. To compensate for these challenges and enhance kinetics at low temperatures, nickel is deposited on specialized supports, such as cerium oxide (CeO_2_) [41]. The use of ceria brings significant benefits, such as a high concentration of oxygen vacancies and considerable basicity [31]. These properties are especially beneficial, since they facilitate the adsorption of acidic CO_2_ molecules on the catalyst surface and promote their subsequent activation [50]. Exploiting these metal-support interactions has yielded some of the most notable results in recent literature. For instance, optimized Ni/CeO_2_ and Ni/TiO_2_ catalysts achieve > 90% CO_2_ conversion at temperatures as low as 250–260 °C under high space velocities (GHSV > 50,000 mL g^−1^ h^−1^) [30,31]. Modern catalyst design heavily relies on maximizing this metal-support interaction (MSI) by utilizing a broader range of reducible or variable-valence oxides, such as MnO or ZrO_2_. A highly significant mechanistic discovery is that the interface configuration, rather than just metal particle size, directly dictates the reaction pathway. Strong interfacial interactions ensure high reducibility and facilitate the formation of abundant oxygen vacancies. These surface defects work synergistically with the active metal sites to efficiently capture and activate CO_2_ via highly reactive intermediates, effectively steering the process toward deep hydrogenation to methane [51]. To further optimize these interfacial dynamics, researchers have demonstrated that the strength of the metal-support interaction in hydrotalcite-derived nickel catalysts can be precisely adjusted through thermal treatment. This thermal tuning induces a phase transformation that stabilizes dispersed nickel active planes and enhances metallic electronic density, facilitating intermediate activation and providing resistance to sintering and coking [52]. Finally, to directly combat the thermal sintering issue mentioned earlier, modern catalyst engineering is increasingly employing non-thermal dielectric barrier discharge (DBD) plasma. Unlike conventional energy-intensive heating, in situ plasma treatment can efficiently reduce nickel precursors to highly dispersed, active metallic Ni^0^ at ambient temperatures. This low-temperature activation perfectly complements systems with strong MSI, yielding catalysts that maintain high methanation activity and long-term stability below conventional reaction temperatures without thermal agglomeration [53]. Interestingly, the traditional methanation preference of nickel can also be completely inverted. Single-atom nickel catalysts can be stabilized against high-temperature deactivation via alkali-mediated dynamic reconstruction under reaction conditions. The in situ formed oxygen-coordinated interface enhances basicity for CO_2_ activation, moderates hydrogen dissociation to suppress methanation, and redirects the reaction via a selective carboxylate route [47].

### 3.3. Bimetallic Catalysts

To optimize costs while maintaining high activity, modern materials engineering is developing bimetallic systems in which doping nickel with a second metal diametrically changes the reaction mechanism. A representative example is Ni-Fe catalysts, where iron plays a dual role in these systems: it increases low-temperature reducibility [42] and promotes surface oxides that facilitate CO_2_ activation, C-O cleavage, and intermediate hydrogenation [43,54]. Consequently, iron-promoted systems exhibit the highest activity in the low-temperature regime (approx. 220 °C). However, at higher temperatures (above 300 °C), the presence of iron becomes problematic. Primarily, it leads to excessive surface oxidation and structural changes in the bimetallic particles, resulting in a drop in activity [43]. Another prominent bimetallic configuration involves alloying nickel with cobalt (Ni-Co). The addition of Co leads to the formation of a solid solution, transferring electrons to neighboring Ni atoms and fundamentally modifying the catalyst’s d-band energy. Due to differences in surface energy, Ni tends to enrich the catalyst surface, ensuring high accessibility of active sites. Furthermore, Ni-Co alloys exhibit distinct resistance to carbon deposition during long-term operation. This tailored bimetallic interface also effectively suppresses undesirable side reactions, such as the RWGS reaction, directing the catalytic pathway toward more valuable products [55]. Furthermore, doping nickel with trace amounts of a noble metal, such as ruthenium, creates highly efficient systems. Ru drastically improves the homogeneous dispersion of active Ni nanoparticles. The key to this enhancement is the hydrogen spillover effect: hydrogen easily dissociates on Ru sites into atomic H species and migrates to neighboring Ni sites. This lowers the reduction temperature and activation energy for CO_2_ methanation. Furthermore, Ru doping promotes electron transfer, maintaining a higher proportion of active metallic Ni^0^ on the surface. Consequently, these bimetallic catalysts achieve high CO_2_ conversion rates at low temperatures, whereas the performance of single-nickel catalysts is limited [56]. The introduction of rare-earth promoters, such as praseodymium, similarly modifies the electronic structure of supported nickel catalysts by promoting a valence transition and generating surface oxygen vacancies. This electronic enrichment stabilizes the bent configuration of adsorbed CO_2_, shifting the mechanism toward direct C-O cleavage to accelerate low-temperature methanation while maintaining structural durability [57]. While the aforementioned systems focus on maximizing methanation efficiency, bimetallic alloying can also be strategically utilized to completely invert the selectivity of nickel. Incorporating an indium core–shell layer over nickel causes electron transfer and negative charge accumulation. The resulting electrostatic repulsion weakens CO adsorption, kinetically blocking methane formation and shifting the pathway toward RWGS [48]. Beyond methanation and RWGS, precise bimetallic engineering at the atomic scale has yielded significant development in direct methanol synthesis. For example, highly optimized bimetallic nickel-tin configurations (Ni_5_Sn_1_/MC) have demonstrated significant activity at low temperatures (175 °C), achieving methanol yields of up to 86.31 mmol g^−1^ h^−1^ under atmospheric pressure [21].

### 3.4. Multimetallic Catalysts

To push the boundaries of catalytic efficiency and structural stability, modern materials engineering is developing complex multimetallic systems in which combining multiple elements optimizes the reaction mechanism [11,44]. A notable example is the application of quinary high-entropy alloys (HEAs), such as the Ni-Co-Fe-Cu-Zn system, designed specifically for high-temperature CO_2_ hydrogenation. In this system, individual metals play highly complementary roles: while oxophilic elements like iron and cobalt provide the primary active matrix for CO_2_ activation, nickel facilitates critical hydrogen dissociation. On the other hand, copper and zinc effectively stabilize oxygen-containing intermediates and isolate active sites to suppress deep hydrogenation, while the high-entropy mixing significantly enhances the overall structural stability of the alloy [44]. Consequently, this five-component multimetallic system achieves considerable turnover frequencies, outperforming commercial nickel catalysts. Furthermore, it exhibits resistance to principal deactivation pathways, such as coking and thermal degradation, maintaining robust performance during long-term continuous operation [37]. Another prominent multimetallic configuration involves tailoring quaternary systems, such as Cu-Zn-Zr-Ga, for the selective hydrogenation of CO_2_ to methanol [45,46]. While binary Cu-Zn is recognized for its high initial activity, it inherently suffers from poor thermal stability and by-product formation under harsh reaction conditions [46]. The addition of elements like zirconium and gallium systematically modifies the catalyst’s electronic properties and increases the concentration of surface oxygen vacancies. This compositional modulation shifts the intermediate binding energies much closer to the optimal thermodynamic value by providing a nearly continuous binding energy distribution, which maximizes the overall methanol yield [44]. Similarly, alloying nickel with specific transition metals to form ternary systems, such as Ni-Co-Fe, creates active and cost-effective catalysts for CO_2_ methanation. In these multi-element frameworks, the synergistic interactions between Ni, Co, and Fe effectively optimize the active sites for hydrogen adsorption and weaken the strong C-O bonds, while maintaining high structural stability [42,50]. Consequently, these tailored multimetallic catalysts achieve high reaction rates, comparable to noble-metal systems, while simultaneously reducing large-scale production costs and enabling scalable commercialization [50,55].

## 4. Conclusions and Perspectives

Catalytic CO_2_ hydrogenation has transitioned from fundamental laboratory research toward practical industrial implementation. It offers clearly defined conversion directions, primarily toward synthetic natural gas (methane), as well as related routes to methanol and sustainable aviation fuels. Recent advancements have successfully pushed the boundaries of low-temperature activity and process efficiency. However, as demonstrated in this review, the transition to full-scale application faces critical bottlenecks. Conventional catalytic systems are frequently limited by inherent thermodynamic constraints and remain highly susceptible to reaction-induced deactivation, particularly coking and byproduct poisoning under continuous operation. Developing precisely engineered materials can overcome these barriers by tailoring atomic-level active site configurations and enabling direct, single-step conversions. Despite these promising strategies, critical research gaps remain. Most current performance evaluations rely on idealized laboratory conditions, highlighting an urgent need for future research to prioritize long-term stability testing under real-world gas feeds containing industrial impurities. Future research and development must specifically prioritize key engineering hurdles: enhancing catalyst stability against sintering, evaluating impurity tolerance in industrial gas feeds, and developing efficient regeneration strategies. Standardized benchmarking, dynamic operation optimization for intermittent renewable energy, and advanced reactor–catalyst integration are crucial for accelerating commercial deployment. Ultimately, the commercial scale-up of these pathways requires a holistic approach that integrates refined catalyst structure–activity relationships with optimized process designs. Achieving a sustainable circular economy will also depend on maximizing overall energy efficiency and securing a cost-effective supply of green hydrogen.

## Figures and Tables

**Table 1:** Standard enthalpies and Gibbs energies (based on [6]).

Product	Reaction	Δ*H*_298 K°_ kJ/mol^−1^	Δ*G*_298 K°_ kJ/mol^−1^
Carbon monoxide	CO_2 (g)_ + H_2 (g)_ → CO _(g)_ + H_2_O _(g)_	41.2	28.6
Methane	CO_2 (g)_ + 4 H_2 (g)_ → CH_4 (g)_ + 2 H_2_O _(g)_	−165	−113
Methanol	CO_2 (g)_ + 3 H_2 (g)_ → CH_3_OH _(g)_ + H_2_O _(g)_	−49	3.83

Note: All thermodynamic values are calculated for standard state conditions (298 K, 1 atm).

**Table 2:** Comparative analysis of representative catalyst categories for CO_2_ hydrogenation, highlighting their main advantages, operational parameters and limitations.

Catalyst Category (Example)	Target Reaction	Key Operating Conditions and Performance	Advantages (Pros) and Structure-Property	Limitations, Cons and Deactivation Behavior	Ref.
Noble Metal (Ru/TiO_2_)	Methanation	Cond: T = 300 °C P = 0.1 MPa H_2_/ CO_2_ ratio = 4 Perf: X ≈ 80% S ≈ 100% (CH_4_) TOS ≈ 16 h	Unique multi-atom surface arrangements (Ru_x_-CO) weaken C-O bonds; high low-temperature activity.	High cost prohibits large-scale industrialization. Deactivation: Prone to thermal sintering.	[38,39,40]
Nickel-based (Ni/CeO_2_)	Methanation	Cond: T = 340 °C (or 250 °C) P = 0.1 MPa H_2_/ CO_2_ ratio = 4 Perf: X ≈ 91.1% (82.5% in 250 °C) S ≈ 100% (94.8% in 250 °C) (CH_4_) TOS ≈ N/R	Strong metal-support interaction via oxygen vacancies boosts CO_2_ activation and stabilizes active sites.	Deactivation: Highly susceptible to coking (carbon deposition) and thermal agglomeration at high temps.	[30,31,41]
Bimetallic (Ni-Fe)	Methanation	Cond: T = 250 °C P = 0.1 MPa H_2_/ CO_2_ ratio = 4 Perf: X ≈ 35% S ≈ 100% (CH_4_) TOS ≈ N/R	FeO_x_ surface species promote direct C-O cleavage; improved low-temp. reducibility.	Deactivation: Susceptible to excessive surface oxidation and structural degradation at T > 300 °C.	[42,43]
Multimetallic (Ni-Co-Fe-Cu-Zn)	Methanation	Cond: T = 300 °C P = 0.1 MPa H_2_/ CO_2_ ratio = 4 Perf: X ≈ N/R S ≈ High (CH_4_) TOS ≈ 100 h	High-entropy matrix physically isolates active sites; robust structural stability.	Complex synthesis. Deactivation: Highly resistant to coking and thermal degradation.	[37,44]
Cu-based (Cu/ZnO/Al_2_O_3_)	Methanol Synthesis	Cond: T = 250 °C P = 3.0 MPa H_2_/ CO_2_ ratio = 3 Perf: X ≈ 23.1% S ≈ 31.2% (CH_3_OH) TOS ≈ 1000 h	Synergistic Cu-ZnO interface drives high selectivity; established industrial baseline.	Deactivation: Highly sensitive to water-induced deactivation and thermal sintering of Cu particles.	[15,19,22]
Bimetallic (Carbon-supported) (Ni_5_Sn_1_/MC)	Methanol Synthesis	Cond: T = 175 °C P = 0.1 MPa H_2_/ CO_2_ ratio = 3 Perf: X ≈ 39.9% S ≈ 88% (CH_3_OH) TOS ≈ N/R	Sn modulates Ni electron density; high yield at ultra-low temperature and atmospheric pressure.	Deactivation: Carbon supports are susceptible to oxidative degradation in water-rich environments.	[10,21]
Multimetallic (Cu-Zn-Zr-Ga)	Methanol Synthesis	Cond: T = 270 °C P = 3.0 MPa H_2_/ CO_2_ ratio = 3 (or 4) Perf: X ≈ N/R S ≈ 100% (CH_3_OH) TOS ≈ N/R	Zr and Ga modify electronic properties; a continuous binding energy distribution maximizes yield.	Complex synthesis procedure. Deactivation: Potential stability issues under harsh conditions.	[45,46]
Supported Metals (Ni-O-K)	RWGS	Cond: T = 500–600 °C P = 0.1 MPa H_2_/ CO_2_ ratio = 1 (or 3) Perf: X ≈ N/R S ≈ High (CO) TOS ≈ N/R	Single-atom interfaces (Ni-O-K) alter surface basicity; kinetically blocks methanation, maximizing CO yield.	Requires high energy input (temp.). Deactivation: Severe risk of coking and agglomeration in H_2_-deficient conditions.	[47]
Bimetallic (Selectivity Inverted) (Ni-In)	RWGS	Cond: T = 250 °C P = 5.0 MPa H_2_/ CO_2_ ratio = 3 (or 4) Perf: X ≈ 12.7% S ≈ 80% (CO) TOS ≈ 12 h	Indium shell electron transfer creates electrostatic repulsion against CH_4_, totally inverting selectivity to CO.	Requires highly precise atomic-level interface engineering.	[48]

Note: T = Temperature; P = Pressure; X = Conversion; S = Selectivity; TOS = Time-on-stream; N/R = Not reported in the original comparative study.

## Data Availability

No new data were created or analyzed in this study. Data sharing is not applicable to this article.
